# Anatomic Osteochondral Allograft Reconstruction for Concomitant Large Hill–Sachs and Reverse Hill–Sachs Lesions

**DOI:** 10.1016/j.eats.2022.08.057

**Published:** 2022-12-21

**Authors:** Rami G. Alrabaa, Ajay S. Padaki, Chaiyanun Vijittrakarnrung, Drew A. Lansdown, Utku Kandemir, Jennifer Tangtiphaiboontana

**Affiliations:** Department of Orthopaedic Surgery, University of California – San Francisco, San Francisco, California, U.S.A.

## Abstract

Glenohumeral instability causing bipolar bone loss is increasingly being recognized and treated to minimize recurrence. Large Hill–Sachs and reverse Hill–Sachs lesions of the humerus must be addressed at the time of surgery to prevent recurrent dislocations and restore the native anatomic track. For patients with epilepsy, locked dislocations may create defects that must be addressed with bony procedures, including osteochondral allograft reconstruction as soft-tissue remplissage may not adequately addresses the magnitude of the bone loss. Osteochondral allografts have been successfully used to address bony defects ranging from 20% to 30% of humeral bone loss whereas shoulder arthroplasty is indicated for larger defects where the native anatomy can no longer be restored. In this Technical Note, we present a technique to address concomitant large Hill–Sachs and reverse Hill–Sachs lesions.

Anterior shoulder instability impacts approximately 1 per 1,000 patients annually in high-risk cohorts, for example, in contact athletes and military patients.^1^ While anterior shoulder dislocations account for greater than 90% of glenohumeral instability, posterior dislocations are increasingly being recognized.^2^ Specific subsets, including those patients with epilepsy, are at increased risk for posterior glenohumeral instability.^3^

While arthroscopic capsulolabral repair for isolated anterior and posterior labral injuries has been described extensively,^4^ recurrent instability with consequent bipolar bone loss often necessitates different treatment options. Posterior dislocations with less than 20% humeral bone loss in reverse Hill–Sachs lesions have been treated successfully with reduction and subscapularis remplissage,^5^ the McLaughlin procedure, or modified McLaughlin procedure. Greater amounts of bone loss may be better treated with osteochondral allograft reconstruction to restore the native anatomy.^6^ Similarly, although arthroscopic labral repair with infraspinatus remplissage has been described extensively for anterior shoulder instability with minimal bone loss,^7^ locked dislocations with large Hill–Sachs lesions also may benefit from osteoarticular reconstruction.^8^ Here, we present a technique for humeral head osteochondral allograft reconstruction for concomitant Hill–Sachs and reverse Hill–Sachs lesions (Video 1).

## Surgical Technique (With Video Illustration)

**Fig 1:**
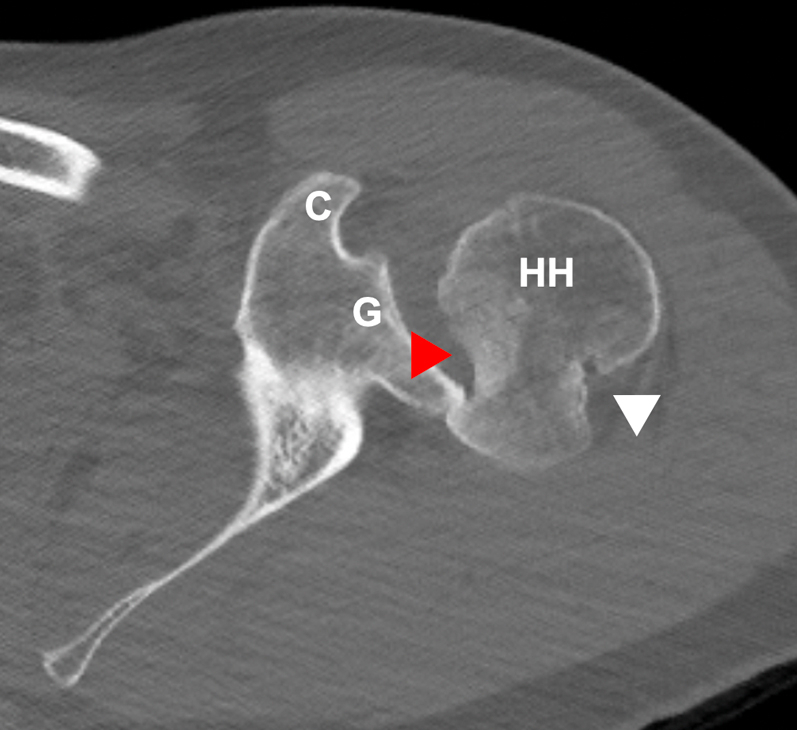
Axial computed tomography image of the patient’s left shoulder showing large Hill–Sachs (white arrowhead) and reverse Hill–Sachs lesions (red arrowhead) with the glenohumeral joint dislocated posteriorly engaging into the reverse Hill-Sachs lesion. (C, coracoid; G, glenoid; HH, humeral head.)

**Fig 2:**
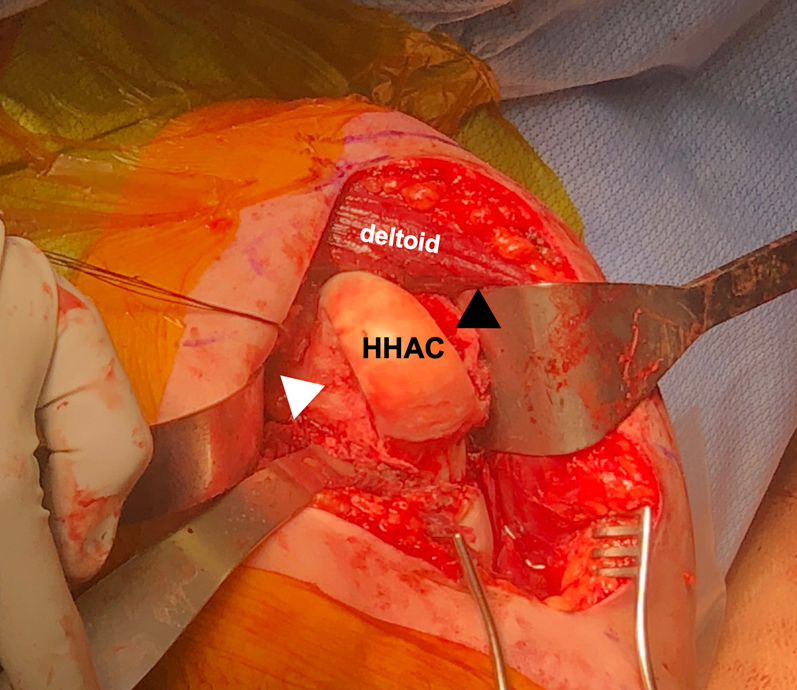
Intraoperative photo of the left shoulder after exposure and dislocation of the humeral head from an anterior deltopectoral approach with a subscapularis peel demonstrating adequate access to the humeral head lesions. The arm is placed in extension, adduction, and maximal external rotation to expose the posterior Hill–Sachs (white arrowhead) and anterior reverse Hill–Sachs lesions (black arrowhead). Only a narrow strip of humeral head articular cartilage (HHAC) remains between the 2 lesions. The Hill–Sachs lesion measured 4 cm × 1 cm × 2 cm and the reverse Hill–Sachs lesion measured 5 cm × 1.3 cm × 2 cm. (HHAC, intact humeral head articular cartilage.)

Preoperative imaging of the patient in this case example shows large Hill–Sachs and reverse Hill–Sachs lesions of the left shoulder without significant glenoid bone loss (Fig 1). A standard deltopectoral approach is performed. The superior one-third of the pectoralis major tendon is released from its insertion on the humeral to aid in exposure. A subscapularis peel is performed by releasing the tendon off of its insertion on the lesser tuberosity. Care is taken to coagulate the anterior humeral circumflex vessels at the inferior border of the subscapularis. The inferior capsule is released from the humeral head to allow for dislocation and adequate exposure of the humeral head so both anterior and posterior lesions can be accessed. The humeral head is dislocated with external rotation, adduction, and extension of the limb. Both the Hill–Sachs and reverse Hill–Sachs lesions should be adequately visualized and accessible at this point before proceeding (Fig 2). If the posterior Hill–Sachs lesion cannot be properly accessed with maximal external rotation, the inferior capsule may have not been adequately released.

**Fig 3:**
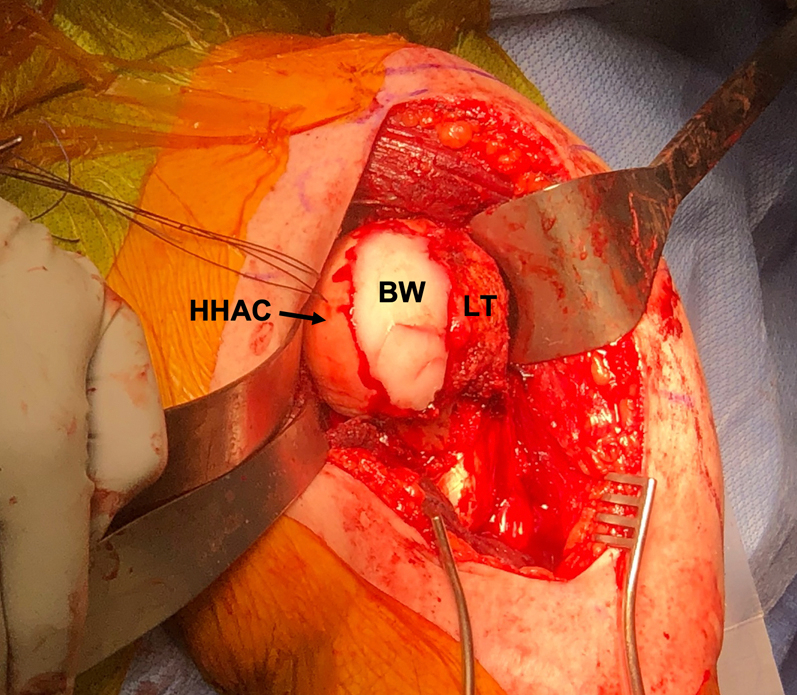
Intraoperative photo of the left shoulder after exposure and dislocation of the humeral head from an anterior deltopectoral approach with a subscapularis peel. The anterior reverse Hill–Sachs defect is first filled with bone wax (BW) to recreate normal anatomy and restore sphericity of the humeral head using the intact humeral head articular cartilage (HHAC) medially and the lesser tuberosity (LT) laterally as guides. (BW, bone wax; HHAC, intact humeral head articular cartilage; LT, lesser tuberosity.)

The lesions on the humeral head are prepared into flat surfaces with a sagittal saw and burr. Bone wax is used to fill each defect to replicate the native anatomy (Fig 3). The anterior reverse Hill–Sachs defect is first filled with bone wax using the medial articular cartilage and lateral lesser tuberosity as guides to judge the appropriate height of the patient’s native humeral head. The bone wax is used as a template to harvest the same size wedge from the frozen proximal humeral osteochondral allograft. On the back table, the bone wax template wedge is measured across all dimensions and the allograft is marked for planned cuts to harvest the same size wedge. Clamps can be used by an assistant to maintain control and stability of the allograft while cuts are made with the sagittal saw.

**Fig 4:**
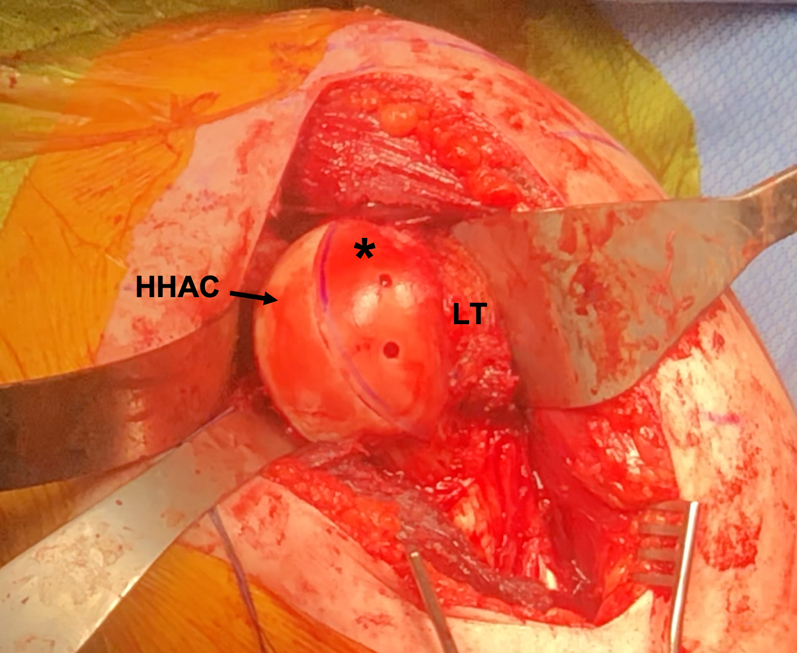
Intraoperative photo of the left shoulder after an anterior deltopectoral approach with an osteochondral allograft wedge (∗) fixed in the reverse Hill–Sachs lesion with 2 headless compression screws. The native humeral head articular cartilage is labeled (HHAC) as well as the lesser tuberosity (LT). The allograft wedge is fixed flush with the native humeral head articular cartilage. The screw tracks are visible with the 3.5-mm headless compression screws embedded and recessed from the articular surface (Acutrak headless compression screw system; Acumed, Hillsboro, OR). (HHAC, intact humeral head articular cartilage; LT, lesser tuberosity.)

**Fig 5:**
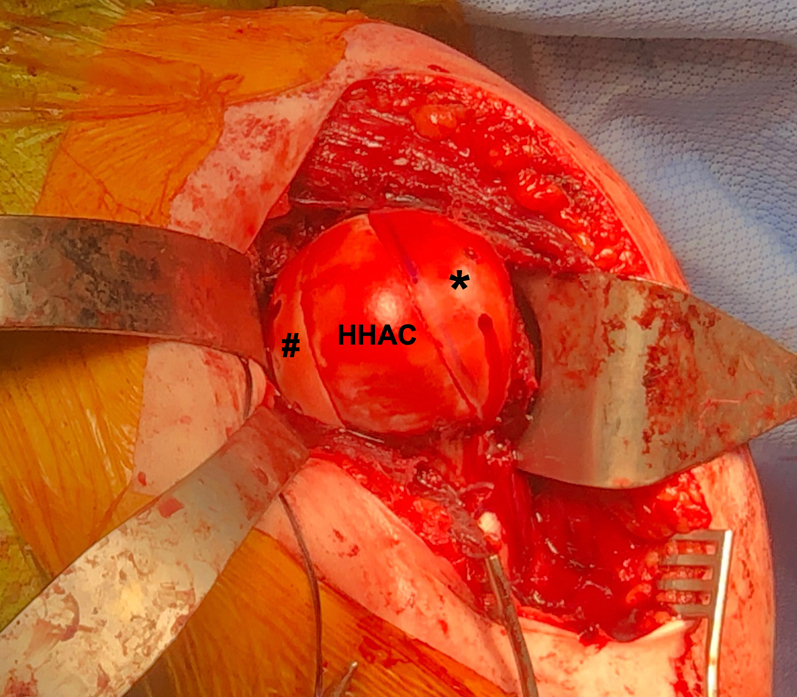
Intraoperative photo of the left shoulder after an anterior deltopectoral approach showing completed fixation of osteochondral allograft wedges in both the reverse Hill–Sachs lesion (∗) and the Hill–Sachs lesion (#). The two osteochondral allograft wedges are anterior and posterior to the native humeral head articular cartilage (HHAC). Both wedges are flush with the native articular cartilage and match the topography well. The headless compression screws are recessed below the articular surfaces of the allograft wedges and are therefore not visible. (HHAC, intact humeral head articular cartilage.)

**Fig 6:**
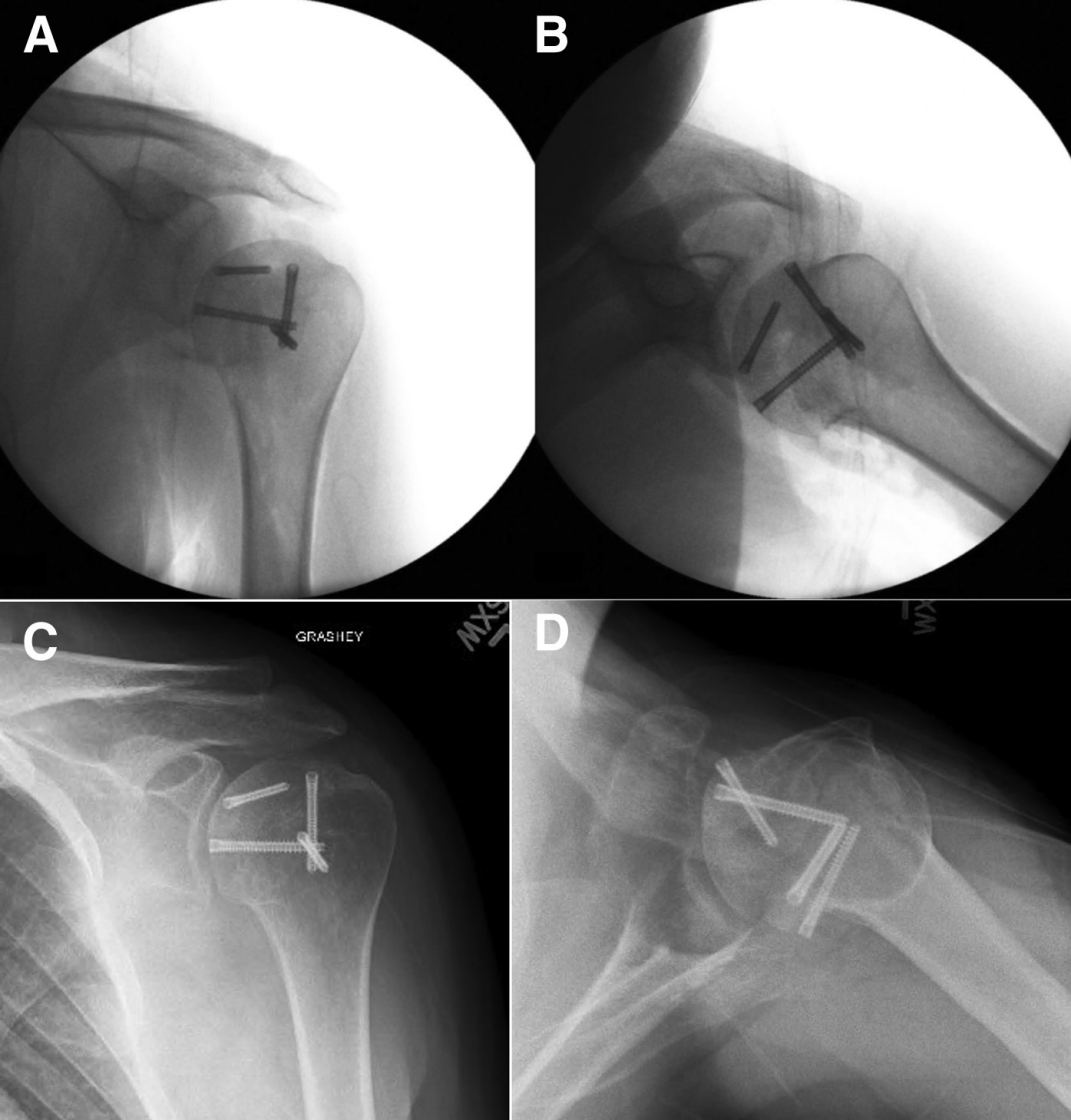
Intraoperative fluoroscopic images and 6-month follow-up radiographs of the left shoulder after completed fixation of osteochondral allograft wedges for reverse Hill–Sachs and Hill–Sachs lesions. (A) Intraoperative anteroposterior image shows the screws recessed below the articular cartilage. (B) Intraoperative axillary lateral view shows restoration of the native humeral head contour and screws recessed below the articular cartilage. (C and D) Six-month postoperative Grashey and axillary lateral radiographs of the left shoulder demonstrating interval healing of the graft without evidence of resorption.

The osteochondral allograft wedge is then provisionally held in place in the anterior reverse Hill–Sachs lesion with 2 k-wires and then definitively fixed with 2 cannulated headless compression screws (Acutrak headless compression screw system; Acumed, Hillsboro, OR). Care is taken to ensure the screw is recessed from the articular surface of the allograft wedge (Fig 4). This process is then repeated for the posterior Hill–Sachs lesion (Fig 5). Intraoperative fluoroscopy can be obtained to confirm restoration of anatomy of humeral head (Fig 6).

After completion of allograft wedge fixation, the subscapularis is repaired. If the subscapularis tendon is not able to reach its insertion onto the lesser tuberosity with relative ease, then the tendon may need to be mobilized from the capsular tissue and middle glenohumeral ligament in order to gain more excursion. Transosseous suture repair is performed by creating three transverse tunnels through the lesser tuberosity with a 2.0-mm drill. A high tensile strength #2 suture is passed through each tunnel and the medial limb of each suture is passed through the subscapularis tendon in modified Mason–Allen configuration. The corresponding suture limbs are tied together completing the subscapularis repair.

Advantages and disadvantages (Table 1), as well as pearls and potential pitfalls (Table 2), of this technique are summarized.

**Table 1:** Advantages and Disadvantages of Humeral Head Osteochondral Allograft Reconstruction for Concomitant Hill–Sachs and Reverse Hill–Sachs Lesions

Advantages	Disadvantages
• This technique restores anatomy and bone stock compared with an arthroplasty procedure in the young patient. • An arthroplasty option can still be pursued in the future if reconstruction fails.	• Fresh-frozen proximal humerus allografts may not be available depending on location or institution. • As with other osteochondral allograft implantation procedures there are potential complications of graft resorption, cartilage loss of the allograft, non-union, and infection.

**Table 2:** Pearls and Potential Pitfalls of Humeral Head Osteochondral Allograft Reconstruction for Concomitant Hill–Sachs and Reverse Hill–Sachs Lesions

Pearls	Potential Pitfalls
• Forward flexion and external rotation of the arm enables access to the inferior capsule for release during exposure. • The allograft wedges should lay flush compared with the native humeral head articular cartilage. • If the subscapularis tendon is not able to be tensioned easily back to the lesser tuberosity for repair, then the tendon may need to be mobilized from the capsular tissue and middle glenohumeral ligament in order to gain more excursion. • While harvesting wedges from the proximal humerus allograft on the back table, clamps can be used by an assistant to maintain control and stability of the allograft while cuts are made with the sagittal saw.	• Not properly identifying and coagulating the anterior humeral circumflex vessels during exposure at the inferior aspect of the subscapularis tendon can lead to excessive bleeding that can be difficult to control. • Inadequate release of the inferior capsule may hinder access to the posterior Hill–Sachs lesion even with positioning the limb in maximal external rotation.

## Discussion

Anterior and posterior glenohumeral instability are among the most common pathologies referred to orthopaedic surgeons.^9^ Various arthroscopic and open treatments have been extensively reported for instability with bipolar bone loss, which have generally demonstrated good patient-related outcomes and low recurrence.^10^

Locked anterior and posterior dislocations with large Hill–Sachs and reverse Hill–Sachs lesions can be treated with shoulder arthroplasty.^11^ However, for young patients, in whom native anatomy restoration is prioritized, treatment with bony augmentation procedures with osteochondral allograft reconstruction has demonstrated favorable outcomes.^12^ Allograft reconstruction can restore the joint to being on-track, thereby reducing risk of subsequent dislocations.^13^ Patients with bipolar bone loss are disproportionately predisposed to greater rates of recurrent dislocation, necessitating distinct treatment paradigms. As bony augmentation treatment options evolve for both anterior and posterior humeral lesions,^14^ the ability of surgeons to treat concomitant anterior and posterior lesions has dramatically increased, although it has not been previously described.

Concerns with extensive allograft reconstruction of the humeral head are lack of healing at the interface of the allograft with the native humerus, as well as the potential for collapse of the articular surface or graft resorption. For the patient described in this Technical Note, radiographs at 6 months have shown no evidence of collapse and do demonstrate incorporation of the allograft (Fig 6). The currently described technique does not limit the potential for future arthroplasty reconstruction options if these adverse outcomes are encountered while offering joint preservation and function in a younger patient.

In this technique, we present an anatomic reconstruction option for the young patient with large concomitant Hill–Sachs and reverse Hill–Sachs lesions to preserve native anatomy. Although shoulder arthroplasty may serve as a treatment option for bone loss, the loss of function and need for eventual revision arthroplasty in young patients present significant problems in this treatment paradigm. Therefore, young patients with significant anterior and posterior humeral bone loss from bidirectional instability may most benefit from attempted restoration of native joint anatomy with this technique.

## Supplementary Data

ICMJE author disclosure forms

Video 1 The patient in this case example has a large Hill–Sachs and reverse Hill–Sachs lesion of the left humeral head. A standard deltopectoral approach is performed. The subscapularis tendon is released via a subscapularis peel directly off of its insertion on the lesser tuberosity directly medial to the long head of the biceps tendon. Adequate release of the inferior capsule is performed to be able to access the humeral head lesions with extension and maximal external rotation. The lesions on the humeral head are prepared into flat surfaces with a sagittal saw. Bone wax is used to fill each defect to recreate native anatomy. The bone wax is used as a template to harvest the same size wedge from the proximal humeral osteochondral allograft on the back table. Two allograft wedges are harvested; one for the Hill–Sachs lesion and the other for the reverse Hill–Sachs lesion. The allograft wedges are fixed into each lesion with 3.5 mm cannulated headless compression screws (Acumed Acutrak headless compression screw system; Hillsboro, OR). The subscapularis is repaired via transosseous suture repair.
